# The carbon footprint of group and save in elective and emergency surgery

**DOI:** 10.1308/rcsann.2024.0073

**Published:** 2025-06-13

**Authors:** AV Robinson, O Moses, JA Bass, V Pegna

**Affiliations:** ^1^ University Hospitals Sussex NHS Foundation Trust, UK; ^2^ Brighton and Sussex Medical School, UK; ^3^ Surrey and Sussex Healthcare NHS Trust, UK

**Keywords:** Operations, Transfusions, Carbon footprint, Sustainability, Group and save

## Abstract

**Introduction:**

Climate change is a significant threat to human health, and surgical care is a major contributor to the carbon footprint of hospital medicine. There is wide variation in perioperative group and save (G&S) blood testing that lacks an evidence base. Eliminating low-value clinical investigations in surgical pathways such as the G&S could lead to significant carbon and cost savings.

**Methods:**

All operations within the trust over a 6-month period and all packed red cell requests made within the same timeframe were analysed retrospectively. Patients were categorised by operation and cross-referenced with transfusion data to determine the transfusion rate of each procedure. The carbon footprint (g CO_2_e) of a single G&S was calculated using a bottom-up approach.

**Results:**

Overall, 15,293 operations and 637 red cell requests were included for analysis. Most transfusions across all operation types occurred after the operation day, and only 36 elective cases required intraoperative transfusions. The carbon footprint of the G&S was calculated at 0.43kg CO_2_e for an inpatient sample, and 7kg CO_2_e for an outpatient sample. Eliminating the second G&S in elective cases with a transfusion rate <1% could save 9 tonnes of CO_2_e per year, the equivalent of 24,000 miles in a passenger vehicle.

**Conclusions:**

Transfusion requirements vary significantly for different operation types. Guidelines surrounding perioperative G&S testing should reflect this, which could save avoidable carbon emissions, cost and resources.

## Introduction

The ongoing climate crisis poses a major threat to human health, and yet healthcare systems are a major contributor to climate change.^1–3^ If global healthcare systems were a country, they would emit more carbon than Japan.^3^ The National Health Service (NHS) of the UK emits 25 million tonnes of CO_2_e per year, representing 4% of national emissions, or nearly 15 million transatlantic flights.^4,5^ Therefore, healthcare systems must take action to reduce their impact on climate change to improve human health and mitigate the ongoing impact.

The NHS in the UK has pledged a carbon neutral service by 2040.^5^ The surgical sector is a resource-intensive area of hospital care, which is why the Royal Colleges of Surgeons and the UK Health Alliance on Climate Change have advised on how to reduce the carbon footprint of surgical pathways.^6,7^ Limiting carbon-intensive, low-value processes are important actions that can be taken now to reduce the carbon footprint of healthcare systems.^6,7^

Blood testing is the highest volume clinical activity undertaken in healthcare.^8^ The group and save (G&S) is a test involving a draw of patient blood, usually venous, and analysis to determine blood group type. In many hospitals, samples are stored refrigerated for several days in the haematology laboratory to enable delayed crossmatching and issuing of blood products. Samples are then discarded by incineration, and if blood units are needed later the test must be repeated. NHS Blood and Transplant was estimated to produce 15,000 tonnes of CO_2_ in 2019, which does not include any of the hospital components (including testing).^9^

Transfusion requirements in the perioperative period are varied depending on the type of surgical procedure and patient factors. The Joint United Kingdom Blood Transfusion and Tissue Transplantation Services Professional Advisory Committee (JPAC) issued guidelines recommending for transfusion to be considered with a perioperative haemoglobin (Hb) count of 80 or less,^10^ yet their only advice on which operations require pre-emptive G&S testing is if blood is going to be required. National Institute for Health and Care Excellence (NICE) guidelines rationalise the use of preoperative blood testing in elective cases based on whether the operation is “major/complex”, “intermediate” or “minor”.^11^ However, these do not include whether patients should have a G&S, so this is much at the discretion of individual trusts and clinicians. Equally, no guidelines are provided in emergency surgery.

Several studies have highlighted the unnecessary variance in G&S testing practices, and that default or routine testing for certain procedures must be revised. This has primarily been in the interest of cost (a G&S costs on average £20 per sample), and resource.^12–16^ However, blood testing also has a carbon footprint, which has yet to be determined for G&S samples.^17,18^ The cumulative impact of G&S testing in surgical patients could be significant and represent a low-value process that would be easy to eliminate without compromise to patient safety.

The aim of this study was to calculate the carbon footprint of G&S testing, and to determine transfusion rates for operations undertaken at our hospital over a 6-month period to highlight procedures where G&S testing can be rationalised.

## Method**s**

### Determining transfusion rates

All packed red cell requests over a 6-month period (29 April 2022 to 1 November 2022) were obtained from the University Hospitals Sussex (East) Trust Transfusion Department. Cases were excluded where the requests originated from a known medical ward, maternity services or paediatric patients. Cases with significant missing data were also excluded.

For each unique administration of blood by the laboratory, the following data were collected from the corresponding patients' medical records: age, operation and confidential enquiry into peri-operative deaths category, preoperative and postoperative haemoglobin, number of G&S taken, and timing of transfusion (preoperative, intraoperative and postoperative).

Data for all surgical operations occurring at all sites within the trust over the same 6-month period were collected from the central information unit and included for analysis. Duplicate procedures were excluded. For the purposes of this study, non-surgical, obstetric, ophthalmological and paediatric procedures were excluded from analysis. Procedures were grouped by type, and transfusion rates were calculated by comparison with the corresponding crossmatch data of the same cohort during the same time frame.

### The carbon footprint of the G&S

All items involved in the collection of blood tests were obtained from ward stock (tourniquet, sample bag, crossmatch EDTA vacuette, safety collection set, ChlorPrep, gauze and paper). Each item was deconstructed, and each constituent part weighed individually. Consumables involved in sample processing were obtained from the laboratory (additional sample cap, testing well). The Department for Business, Energy and Development Strategy (BEIS) and Inventory of Carbon and Energy databases were used to determine the carbon footprint for individual components. At our trust, samples were analysed using ORTHO VISION^™^ analysers (Ortho Clinical Diagnostics) and stored in Labcold Large Capacity blood bank refrigerators for 7 days.

The carbon footprint of clinical waste disposal in this study was calculated using the figures for clinical waste high-temperature incineration by Rizan *et al* (1,074kg CO_2_e/tonne including transport), which was undertaken at the host institution.^19^ See system boundary in Appendix 1 for further details.

An audit of 29 patients presenting for laparoscopic cholecystectomy preoperative assessments found an average round trip of 23 miles per appointment. Because the second G&S blood test requires a separate attendance by the patient within 7 days of the procedure, the carbon cost for transport is a necessary included factor for elective patients. The BEIS database value of 0.283kg CO_2_e/mile was used (medium sized car, unknown fuel type) and hence 6.56kg CO_2_e per second test. The full methodology can be found in Appendix 2.

## Results

**Figure 1:**
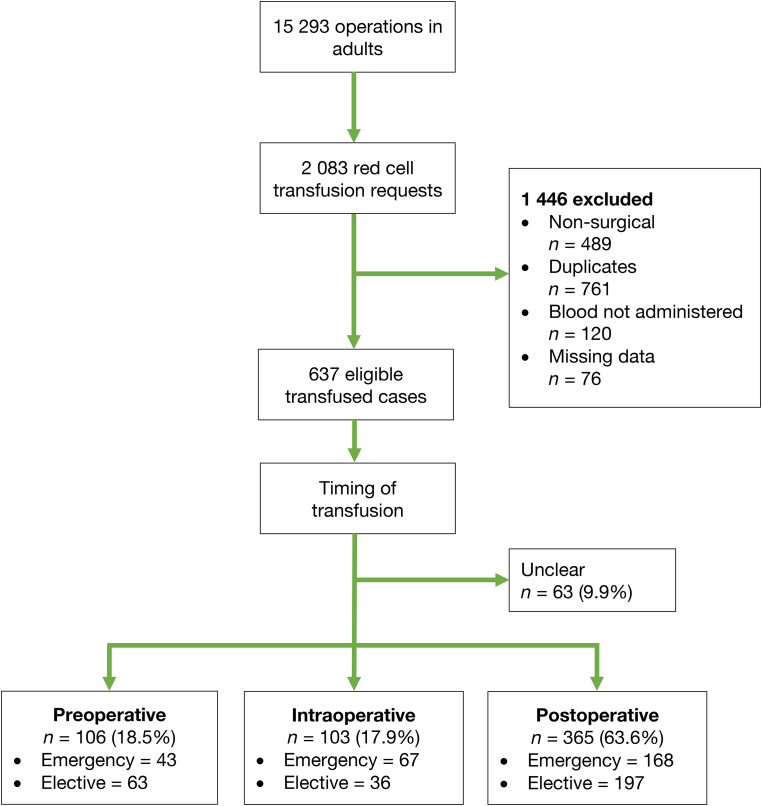
Patient flow diagram

A total of 15,293 operations were performed in adults between 29 April 2022 and 1 November 2022. Transfusion records for 2,083 blood requests were reviewed; 1,446 were excluded because they were non-surgical patients (489), duplicates (761) or had significant missing data (76), or the units of blood were not administered (120). This left 637 cases for analysis (Figure 1).

Of the 637 transfused cases alone, there were 1,536 G&S tests sent (mean 2.41 per patient), with 401 tests ordered more than requirement (already a valid sample in storage for crossmatching). Transfusions occurred preoperatively in 106 cases (18.5%), intraoperatively in 103 cases (17.9%), postoperatively in 365 (63.6%) cases, and in 63 cases the exact timing of transfusion was unclear (Figure 1).

**Figure 2:**
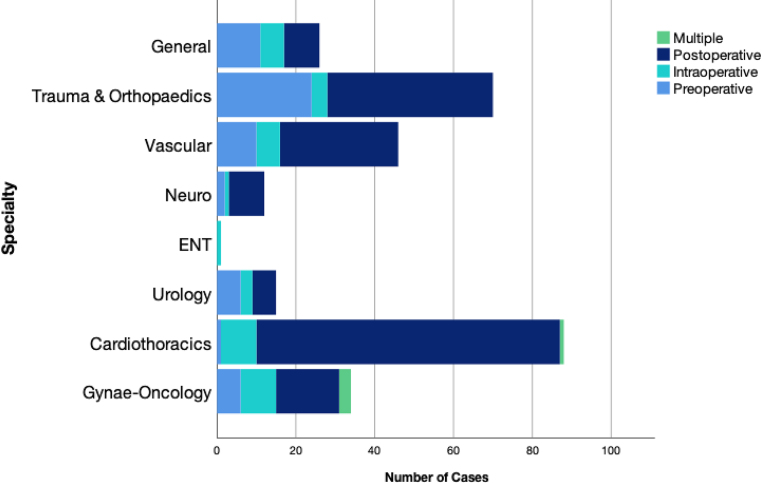
Transfusion timing for elective case

**Figure 3:**
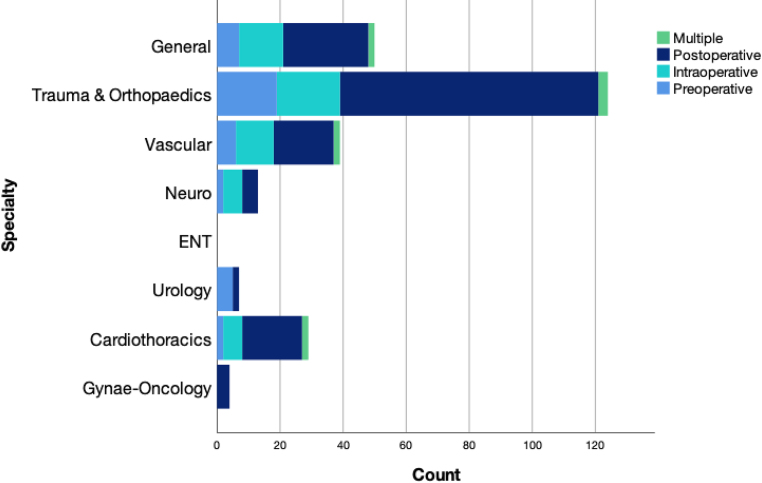
Transfusion timing for emergency cases

Overall perioperative transfusion rates for each specialty are displayed in Table 1. By contrast, day-of-surgery transfusions are seen in Table 2 and are comparatively low. The most transfused procedures are listed in Table 3, vs the most performed procedures over this period in Table 4. Cardiothoracic surgery represented the specialty with the highest intraoperative transfusion rate (8.05%), followed by vascular surgery (2.89%) and neurosurgery (1.38%). The timing of transfusion is represented by Figures 2 and 3, wherein it can be appreciated that most transfusions occur in the postoperative phase (after the day of operation), which is common for both elective and emergency procedures. These data demonstrate that overall, there is some predictability in cases that will require intraoperative transfusion and many cases could avoid unnecessary testing preoperatively. Further, only 36 of the 103 intraoperative transfusions occurred in elective cases, the details of which are listed in Table 5. Operations requiring day-of-surgery transfusions are available in Appendix 3.

**Table 1:** Summary overall transfusion rates for major specialties during time frame

	Elective		Emergency		Intraoperative (electiveand emergency)
	Total procedures	Transfused (%)	Total procedures	Transfused (%)	
General surgery	748	17 (2.27)	855	53 (6.20)	21 (1.31)
Trauma and orthopaedics	1,490	31 (2.08)	1,779	176 (9.89)	24 (0.73)
Vascular	430	50 (11.63)	262	42 (16.03)	20 (2.89)
Neurosurgery	231	14 (6.06)	277	15 (5.42)	7 (1.38)
Otolaryngology	806	1 (0.12)	27	0 (0)	1 (0.12)
Urology	1,052	14 (1.33)	148	7 (4.73)	3 (0.25)
Cardiothoracics	147/174 (84.5)				14 (8.05)
Maxillofacial	231	0 (0.00)	7	0 (0.00)	0 (0.00)

**Table 2:** Day-of-surgery transfusions per specialty, *n* (% of cases within category) (%)

	Elective	Emergency	Total
General	6 (0.80)	17 (1.99)	23 (1.43)
Trauma and orthopaedics	11 (0.74)	48 (2.70)	59 (1.80)
Vascular	14 (3.25)	18 (6.87)	32 (4.62)
Neurosurgery	2 (0.86)	8 (2.89)	10 (1.96)
Otolaryngology	1 (0.12)	0	1 (0.12)
Urology	3 (0.29)	4 (2.70)	7 (0.58)
Cardiothoracics	72 (41)		
Gynae-oncology	12	1	13
Total			217

**Table 3:** Most commonly transfused operations during observed period

Procedure	Transfused (*n*)	Total (*n*)	Transfused (%)
Open cardiac surgery (elective or emergency)	138	147	93.88
Amputation of ischaemic limb (elective or emergency)	42	85	49.41
Abdominal wall abscess	3	8	37.50
Elective revision major joint replacement (hip, knee, shoulder, elbow)	16	49	32.65
Emergency laparotomy	48	167	28.74
Elective upper gastrointestinal resection for cancer	3	11	27.27
Elective iliac/femoral/popliteal bypass grafting	19	82	23.17
Emergency femoral nail for neck of femur fracture	48	219	21.92
Emergency infected joint /metalwork management	16	73	21.92
Open abdominal aortic aneurysm repair (elective or emergency)	6	39	15.38
Emergency arthroplasty for neck of femur fracture	35	254	13.78
Elective lower gastrointestinal bowel resection	11	91	12.09
Emergency intracranial haematoma evacuation	7	58	12.07
Elective craniectomy/craniotomy + proceed (including excision of intracranial lesion)	5	54	9.26
Emergency fixation of femur or tibia (not neck of femur fracture or ankle fracture)	29	395	7.34
Emergency hip fracture fixation	5	76	6.58
Spinal decompression/fusion (elective or emergency)	8	123	6.50
Elective transurethral resection of bladder tumour	5	105	4.76
Revision/removal of metalwork (pelvis/long bones)	3	100	3.00
Major joint replacement (hip, knee, shoulder, elbow)	8	314	2.55

**Table 4:** Perioperative transfusion rates for most commonly performed operations requiring preoperative group and save in observed period

	Transfused (*n*)	Total (*n*)	Transfused (%)
Elective			
General			
Laparoscopic cholecystectomy	1	135	0.74
Groin hernia repair	0	111	0
Abdominal wall hernia repair	0	98	0
Lower gastrointestinal bowel resection	11	91	12.09
Trauma and orthopaedics			
Total hip replacement	7	141	4.96
Knee replacement (total or unicompartmental)	1	133	0.75
Urology			
Cystoscopy ± proceed	4	300	1.33
Ureteroscopy ± proceed	1	197	0.51
Transurethral resection of bladder tumour	5	105	4.76
Transurethral resection of prostate	1	88	1.14
Emergency			
General			
Appendicectomy	1	242	0.41
Laparotomy	48	167	28.74
Abscess incision and drainage	3	117	2.56
Hernia repair (including laparotomy)	1	55	1.82
Trauma and orthopaedics			
Wound examination/debridement/closure	11	311	3.54
Upper limb fracture fixation	6	280	2.14
Hip replacement for neck of femur fracture	35	254	13.78
Femoral nail/fracture fixation	48	219	21.92
Foot/ankle fracture fixation	3	165	1.82
Vascular			
Foot debridement ± toe amputation	5	95	5.26

**Table 5:** Intraoperative transfusions in elective surgical cases

	*n*
Open heart surgery (e.g. bypass grafting, valve replacements)	10
Major gynae-oncological resection (e.g. total abdominal hysterectomy, bilateral salpingoopherectomy, bowel resection, urological resection)	8
Lower gastrointestinal tract resection (e.g. Hartmann’s, hemicolectomy)	3
Above/below knee amputation	2
Cystoscopic resection/laser to bladder tumour	2
Complex total hip replacement	2
Femoral endarterectomy/bypass	2
Panendoscopy	1
Open aortic aneurysm repair	1
Laparoscopic cholecystectomy	1
Major joint arthroplasty (hip, knee, shoulder)	1
Nephroureterectomy	1
Endovascular aortic aneurysm repair	1
Oesophagectomy	1
	36

### Carbon footprint of G&S

The carbon footprint of the G&S was estimated at 0.43kg CO_2_e, comprising 0.15kg CO_2_e in equipment, 0.22kg CO_2_e in laboratory processing and storage and 0.06kg CO_2_e in disposal (see Appendix 2). Hence, excess G&S testing in patients who required a transfusion alone was responsible for 172.43kg CO_2_e (the equivalent of 440 miles in a petrol car), not including patient travel. In the sample of elective cholecystectomy patients, the average journey was 23 miles. Therefore, one outpatient sample alone could be responsible for up to 7kg CO_2_e.

In elective cases where the transfusion rate was <1% and G&S is currently mandated, eliminating the second G&S would save 9,436kg CO_2_e over a 1-year period, the equivalent of over 24,000 miles driven in a passenger vehicle.

## Discussion

This observational study has highlighted operations that commonly require perioperative blood transfusions. We have also demonstrated that many commonly performed procedures (appendicectomy, laparoscopic cholecystectomy, endourological procedures, etc.) are low risk for intraoperative blood transfusion. The carbon footprint of the G&S has been calculated, which can be used as a currency for informing guidelines.

We found that G&S testing occurred in excess of requirements, including at least 401 unnecessary tests in patients who underwent transfusion alone. This may be due to theatre cancellation, poor staff knowledge about timings for valid samples and failing to check historical records. Fortunately, our trust often identifies duplicate requests and does not process them, meaning only the consumable and energy cost up to the point of the laboratory is lost. However, the impact on the patient and resource waste needs to be highlighted. Better understanding of the indications for G&S is warranted to prevent unnecessary harm to patients and consumable and financial waste.

The most commonly transfused specialties on the day of surgery were cardiothoracics (41%), vascular (4.62%) and neurosurgery (1.96%), but with significant variation depending on procedure. In most cases, transfusions occurred after the first postoperative day so the second test could have been postponed and requested only when clinically indicated. Cases at our trust that currently mandate preoperative G&S testing but do not have a significant demonstrable risk of intraoperative transfusion include transurethral resection of the prostate (TURP), laparoscopic cholecystectomy, laparoscopic appendicectomy and hernia repairs. Rationalising the testing in these cases may be advisable, especially where it demands patients attending more appointments to obtain them because travel significantly contributes to the carbon footprint of testing in elective patients.

We have calculated the carbon footprint of the G&S blood test in both an inpatient and outpatient setting, at 430g CO_2_e and 7kg CO_2_e, respectively. The phlebotomy equipment and vacuette container were responsible for 150g, which is comparable to a recent study based in Canada, which calculated 150g for the phlebotomy equipment and up to a further 34g for the vial.^18^ Our study and the Canadian study report higher values for testing overall than McAlister *et al* based in Australia.^17^ However, in all these pathology tests most of the footprint was accountable to sample collection consumables. In the Canadian study, the laboratory testing was calculated to be much lower than that of the G&S as calculated in this study, with tests ranging from 2.2 to 80.8g CO_2_e. Here, we found the laboratory processing was much higher at 220g CO_2_e. The difference may be explained by the need to store the G&S vacuettes in a refrigerator for 7 days. In any case, all studies highlight the need to reduce unnecessary low-value over-investigation.

There has been a wealth of studies identifying the financial and social implications of excess G&S testing. One French national study compared G&S testing in patients undergoing one of four operations – laparoscopic cholecystectomy, lumbar discectomy, thyroidectomy and breast cancer resection – for which their national guidelines do not recommend preoperative G&S. Despite this, up to 50% were still receiving testing costing millions of euros.^16^ Al-Musawi *et al* reviewed 1,891 laparoscopic appendicectomy and hernia repairs and found only one patient requiring a transfusion postoperatively. The cost of the G&S tests in this cohort was almost £50,000 (£13.50 per sample). Barrett-Lee *et al* report only one transfused case in 562 emergency laparoscopic procedures, which was in a patient with a perforated duodenal ulcer with concurrent sepsis and metabolic acidosis.^13^

For commonly performed urological procedures such as TURP and transurethral resection of bladder tumour (TURBT), variation in clinical risk of transfusion has been suggested depending on case factors such as prostate size, tumour size and invasion, and preoperative haemoglobin.^20,21^ Our cohort in this study did not see any intraoperative transfusions for patients undergoing TURP or TURBT, and overall day-of-surgery transfusions were minimal. As such, a case-based approach may be indicated given that these factors can be predicted and assessed preoperatively. Further, day-of-surgery G&S could be considered if necessary to reduce preoperative patient travel.

Within orthopaedics, although a number of patients receive perioperative transfusions overall, the number that occur on the day is low (0.74% for elective and 2.70% for emergencies) and earlier studies have identified no intraoperative or on-the-day transfusions whatsoever in elective total hip and knee arthroplasties, and shoulder arthroplasties.^22–24^ We found only one case requiring intraoperative transfusion in primary joint arthroplasty of 314 cases (0.32%).

Although there is no significant direct harm in over-using the G&S test for patients, it is expensive, carbon-intensive and for elective patients requires two attendances at hospital in the preoperative work-up. Operations such as major joint arthroplasties, TURBT and TURP are common in frail patients and so attending multiple appointments can incur a financial and social burden to carers and health and social care systems. Iatrogenic anaemia due to blood testing has a noticeable cumulative impact within critically ill cohorts, which must not be overlooked.^25,26^ Some hospitals can perform a second test on admission when required,^24^ but this is not the process at the host institution, again adding unnecessary greenhouse gas emissions to the perioperative pathway. For emergency surgical care, the G&S is liberally used without concrete evidence about what acute procedures require them. Real-world transfusion rates for commonly performed operations are reported inconsistently and this is a barrier to informing guidelines on where testing is required.

## Study limitations

It is likely that our carbon footprint estimate is conservative, because we have not performed a life cycle analysis and we were unable to obtain emissions data on reagents. Because we are suggesting reduction or elimination of testing versus current practices, our analysis serves as a reasonable carbon estimate.

The transfusion rates were calculated using coded data from the central information unit and rely on accurate data input. In some cases, such as in cardiothoracic surgery, elective and emergency procedures were not coded so have been grouped. This does not significantly impact the findings of this paper, because we highlight potential areas for more investigation and an evidence-based approach to testing guidelines. In addition, the differential between O-negative blood administration and crossmatched blood could not be provided. It would have been interesting to ascertain what percentage of intraoperative or on-the-day transfusions in fact used O-negative blood and therefore negated the need for the G&S. However, in our trust most electronically issued blood can be suppled within 10 minutes and so O-negative use is reducing.

The outpatient G&S calculation is based on all patients driving to their appointment, because in our locale this represents the most common transport modality to appointments based on a local survey on transport method. To generalise these results, the local impact of patient transport should be considered.

## Conclusions

Surgical pathways urgently need reform to address unnecessary variation in practice and low-value diagnostics. Rationalising the use of G&S blood tests could have a significant impact on the carbon footprint, financial and social cost of surgical care, particularly in elective and day-case procedures. We recommend an evidence-based approach to perioperative G&S guidelines, particularly in emergency surgery where the majority of intraoperative transfusions occur.
